# Supplementary material for: Temporal system-level organization of the switch from glycolytic to gluconeogenic operation in yeast
Source: Mol Syst Biol. 2013 Apr 2;9:651. doi: 10.1038/msb.2013.11 (PMC3693829; doi:10.1038/msb.2013.11)
Supplement: Supplementary File 3 — Supplementary Information and Supplementary Figures S1-3 [file msb201311-s3.pdf]

Supplementary information

Temporal system-level organization of the switch from glycolytic to gluconeogenic operation in yeast

Guillermo G. Zampar[1], Anne Kümmel[2], Jennifer Ewald[2], Stefan Jol[2], Bastian Niebel[1], Paola Picotti[2], Ruedi Aebersold[2,3], Uwe Sauer[2], Nicola Zamboni[2], Matthias Heinemann[1,2]

[1] University of Groningen, Groningen Biomolecular Sciences and Biotechnology Institute, Molecular Systems Biology, Nijenborgh 4, 9747 AG Groningen, The Netherlands

[2] ETH Zurich, Institute of Molecular Systems Biology, Wolfgang-Pauli-Str. 16, 8093 Zurich, Switzerland

[3] Faculty of Science, University of Zurich, Rämistrasse 71, CH-8006 Zurich, Switzerland

Corresponding author

Matthias Heinemann (m.heinemann@rug.nl) +31 50 363 8146

3.1 Changes in cellular carbon content

In this section we describe how we dealt with the variation of cellular composition. During the diauxic shift cells are exposed to a constantly changing environment that could be translated into differences in cellular composition as the adaptation process occurs. In fact, it has been reported that the composition of cells varies with cultivation conditions (Albers *et al.* 2007), so the utilization of fixed equation for biomass production in the stoichiometric model would lead to systematic errors in the estimations of intracellular fluxes.

The variation in the carbon content of cells becomes obvious from the imbalance in the amount of carbon that is taken up and excreted, when a constant cellular carbon content was used (Albers *et al.* 2007) (Figure S1). The most important carbon imbalance occurs as glucose concentration drops, which accounts for the observed decrease in carbon dioxide production right before glucose depletion, while glucose uptake rate remains high (Fig. 1C and H). This imbalance can only be due to a change in cellular carbon content. Indeed, several studies have shown that yeast cells accumulate glycogen, the main reserve carbohydrate in yeast, right before glucose exhaustion and trehalose, which is believed to a act as a stress protectant, immediately after the depletion of glucose (Lillie *et al.* 1980; Parrou *et al.* 1999; Francois *et al.* 2001).

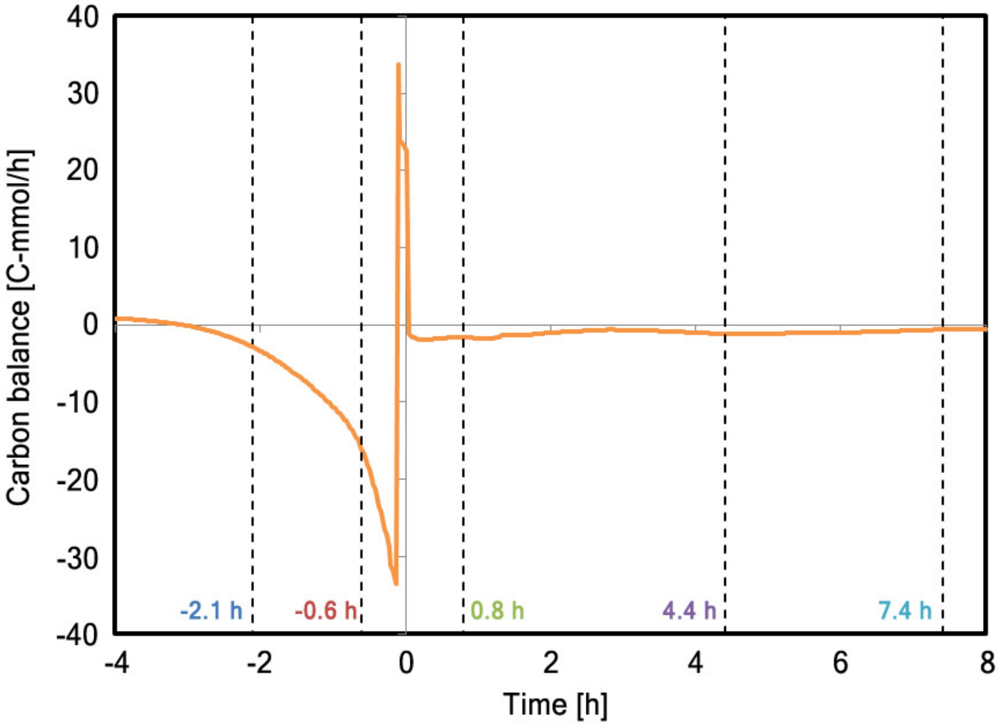

Figure S1: Carbon balance calculated on the basis of the estimated uptake/excretion rates and growth rate as a function of time. For this calculation, a constant cellular carbon content was assumed (taken from Albers *et al.* (2007)).

To capture these changes in cellular carbon content, we included in the stoichiometric model the reactions for trehalose and glycogen biosynthesis (and degradation), and an 'exchange reaction' for each of these compounds (see Supplement 4). Exchange reactions in the context of flux balance analysis, are a technical measure to allow for imbalances. In this way, we are able to use a constant biomass equation while the rate of production (or consumption) of reserve carbohydrates is determined by the experimental and stoichiometric constraints alone.

3.2 Production and utilization of storage carbohydrates

The extended model, including the reactions of trehalose and glycogen biosynthesis and degradation, allowed us to estimate the flux from and towards reserve carbohydrates. However, the inclusion of both carbohydrates (trehalose and glycogen) results in too broad flux ranges because carbon can cycle among these two compounds: i.e. increased glycogen degradation leads to higher production of trehalose, and vice versa.

Instead, much narrower ranges can be obtained for the net flux from/towards total reserve carbohydrates, so we performed the flux variability analysis also with a lumped reaction referred as "Storage". This reaction indicates the amount of glucose residues that are produced or consumed due to the flux from or towards both trehalose and glycogen. A positive value through this reaction means that storage carbohydrates are being accumulated, while negative fluxes indicate a net degradation of storage carbohydrates.

The flux variability analysis through the "Storage" reaction reveals that there is a significant net accumulation of reserve carbohydrates right before glucose depletion (at t = -0.6 h) (Fig. 3F, "Storage"). This result is consistent with previous studies that report that glycogen cellular content increases steeply during the last hour prior to glucose exhaustion (Lillie *et al.* 1980; Parrou *et al.* 1999; Francois *et al.* 2001).

Interestingly, even though glycogen cellular content decreases after glucose depletion (Lillie *et al.* 1980; Parrou *et al.* 1999), our results indicate that there is no net consumption of reserve carbohydrates at t = 0.8 h nor at t = 4.4 h, as revealed by the non-zero minimal flux through the "Storage" reaction (Fig. 3F). Thus, glucose produced by glycogen phosphorolysis is probably used to synthesize trehalose instead of being diverted into glycolysis, which also accounts for the observed increase in trehalose content after depletion of glucose (Lillie *et al.* 1980; Parrou *et al.* 1999). This observation strongly suggests that, contrary to what is generally believed, glycogen is not accumulated for its later utilization as a carbon source but its accumulation during the late glucose phase (just before the diauxic shift) is rather a consequence of the limitation in the glycolytic flux before glucose depletion.

3.3 Pyruvate and acetate utilization

An interesting feature of the diauxic shift is the immediate pyruvate consumption that occurs simultaneous to ethanol consumption directly after glucose depletion (Fig. 1G and S2, "Pyr_EX"). Thus, right after glucose depletion cells present a higher overall nutrient uptake rate as compared to later time points, where only ethanol is consumed.

To get insights into the relevance of pyruvate utilization during this early stage, we further analyzed the differences in metabolic flux distributions between t = 0.8 h and t = 4.4 h. Interestingly, the increased rate of nutrient uptake at t = 0.8 h is not translated into higher fluxes through the TCA or glyoxylate cycles (see for example ACONT reaction in Fig. 3F) but it is rather

redirected towards acetate excretion (Fig. 1B and S2, “Ac_EX”). Thus, during this phase, a significant amount of the taken up ethanol and pyruvate is only partially catabolized.

The production of acetate from ethanol and pyruvate yields two and one moles of NADH per mole of substrate, respectively. Still, if acetate were diverted into the TCA cycle instead of being excreted the output of ATP production would increase significantly. The question is why the cells excrete part of the taken up carbon source as acetate instead of using it to produce more ATP in the TCA cycle? Could it be that cells simply do not need this extra ATP?

Surprisingly, we found that the ATP turnover per growth rate unit is increased drastically at 0.8 h as compared to exponential growth on glucose (Fig. S2, “ATPS”; compare t = -2.1 h and t = 0.8 h). This high ATP turnover, according to our results, is met by an increased production of NADH when acetaldehyde is produced form ethanol (ALCD reaction) and then transformed to acetate (ALDD reaction) (Fig. 3F, “ALDD(m+c)”). The NADH produced in this way is later oxidized in the respiratory chain (Fig. S2, “CYOOm”). Thus, cells rely on the conversion of ethanol and pyruvate to acetate for producing NADH rather than in the TCA or glyoxylate cycles. This effect must be due to either a kinetic or thermodynamic limitation of the TCA/glyoxylate cycle, and it resembles the preference of some cells to use more inefficient pathways akin to fermentation over respiration under high substrate uptake conditions (Crabree effect).

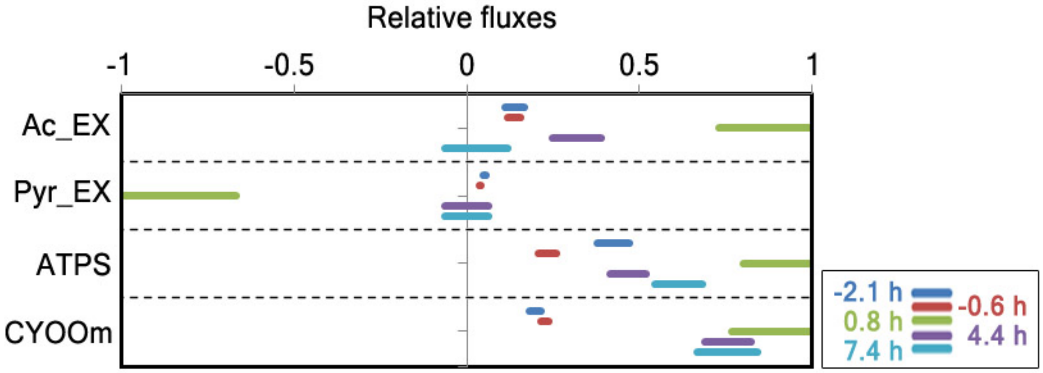

Figure S2: Estimated flux ranges through the reactions indicated relative to the growth rate at each time point. Fluxes were scaled to fit the interval between -1 and 1. For the actual values see Supplement 5.

The higher ATP turnover immediately after glucose depletion is probably related to a higher requirement of ATP for synthesizing the enzymes that are needed for the change in substrate conditions. In fact, the protein expression changes dramatically upon glucose depletion. There is a significant drop in levels of proteins that are involved in the Embden-Meyerhoff pathway and the PP pathway, while enzymes of the TCA cycle, glyoxylate and dicarboxylate metabolism are being up-regulated (Fig. 4A). Further, the expression level of alcohol dehydrogenase 1 (ADH1, the enzyme responsible of ethanol production from acetaldehyde (Bennetzen *et al.* 1982) is strongly down regulated, while the enzyme that catalyzes the reverse reaction, alcohol dehydrogenase 2 (ADH2) (Russell *et al.* 1983), is significantly increased (Supplement 5). These changes in protein abundances most likely account for the increased ATP requirements observed immediately after glucose depletion.

3.4 Predicted transcription factors involved

The over-representation analysis allowed us to identify possible transcriptions factors involved in the diauxic shift on the basis of the changes in protein abundances. Even though our predictions are well-founded by previously reported results (see main text), we searched for further experimental evidence of such involvement. In order to do so, we constructed deletion mutants for each predicted transcription factor (except for Hsf1 and Abf1 which present a lethal phenotype) and analyzed their ability to grow through the diauxic shift. We found that, even though most of the mutants present a growth rate similar to the wild type in the presence of glucose (the only exception being the Ino2 mutant which present a very low growth rate even in glucose, see Supplement 7), once it is depleted they perform less efficiently in terms of biomass production, i.e. they grow slower that the wild type or they do not grow at all after glucose depletion (Figure S3). This clearly indicates that the absence of these transcription factors is detrimental to the adaptation process, which strongly support their participation in regulating the expression of key enzymes during the shift (see main text).

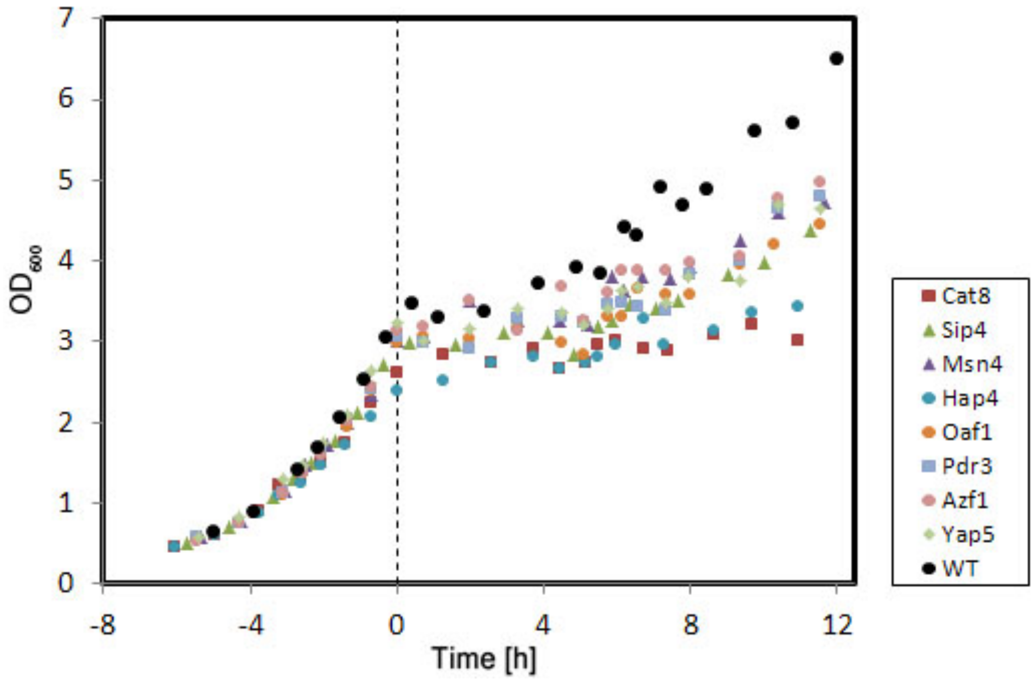

Figure S3: Growth curves corresponding to the wild type strain (WT) and the deletion mutants of the indicated genes. Cells were cultivated in minimal medium supplemented with 5 g/l glucose and optical densities (600 nm) were measured at different time points throughout the late glucose phase and diauxic shift. Time zero indicates the end of exponential growth phase. For the complete data set see Supplement 7

Supplementary methods:

Deletion mutants

The deletion mutants were constructed in the FY4 strain by homologous recombination using a DNA fragment with the G418-resistance gene and a flanking region of about 250 nucleotides to either side of the target gene. This fragment was obtained by PCR amplification using the genomic DNA from the corresponding strain in a deletion mutant library. Transformants were selected using YPD plates supplemented with 200 µg/ml G418. The sequences of the forward (F) and backward (B) primers for each gene are: Yap5F, TTT AGT TTA CCT ATT GGG CCG; Yap5B TCA GCT CAA TTC GTC GGA AA; Ino2F, CCA ACC TAA CCT TTT TTC AGC; Ino2B, TAC TTC ACC ATC ATT GCC TCC; Pdr3F, AAC AAC AGG CGC TGC CCT T; Pdr3B, CTA CTG AAC AGC TGC ATT CCA; Oaf1F, GAA AAC CGC TCA AAA AGG TG; Oaf1B, TTT GCA GCA AGA GGC GAT AT; Azf1F, ACG AAT TGG TGA TCC CAA GA; Azf1B, CCG TCG ATA GAA ATT GAA CCT; Msn4F TGC ATG AGC ATA CCT GTA GGT; Msn4B, CTG CCA AAT GGA AAA AAC GG; Hap4F, TTC ACC TCT CTA AAC CCC AGT; Hap4B, CCT TCC AGA CCC ATA TTT GTT; Sip4F, TTT AAG GGC GTG ATC TCA GAA; Sip4B, TTT TTG GAT GGA CGG GAA TG; Cat8F, CGG CGG GGC TGA TAT TTT; Cat8B, TGT CAG ATG GTC CTC CGG TAA. The mutations were confirmed by determining the presence and correct size of the corresponding amplicon when performing two separate PCRs, one with the forward and backward primers of each gene, and the other one with the forward primer of each gene and the KanB primer. The latter primer anneals within the resistance gene and its sequence is CTG CAG CGA GGA GCC GTA AT.

References

Albers E, Larsson C, Andlid T, Walsh MC, Gustafsson L (2007). Effect of nutrient starvation on the cellular composition and metabolic capacity of Saccharomyces cerevisiae. *Appl Environ Microbiol* **73**: 4839-4848.

Bennetzen JL, Hall BD (1982). The primary structure of the Saccharomyces cerevisiae gene for alcohol dehydrogenase. *J Biol Chem* **257**: 3018-3025.

Francois J, Parrou JL (2001). Reserve carbohydrates metabolism in the yeast Saccharomyces cerevisiae. *FEMS Microbiol Rev* **25**: 125-145.

Lillie SH, Pringle JR (1980). Reserve carbohydrate metabolism in Saccharomyces cerevisiae: responses to nutrient limitation. *J Bacteriol* **143**: 1384-1394.

Parrou JL, Enjalbert B, Plourde L, Bauche A, Gonzalez B, Francois J (1999). Dynamic responses of reserve carbohydrate metabolism under carbon and nitrogen limitations in Saccharomyces cerevisiae. *Yeast* **15**: 191-203.

Russell DW, Smith M, Williamson VM, Young ET (1983). Nucleotide sequence of the yeast alcohol dehydrogenase II gene. *J Biol Chem* **258**: 2674-2682.
